# Flebolitos en la región maxilofacial: un desafío para el diagnóstico por imágenes. Una revisión

**DOI:** 10.21142/2523-2754-0904-2021-086

**Published:** 2021-12-09

**Authors:** Jorge Luis Becerra-Heredia, Gustavo Adolfo Fiori-Chíncaro, Ana María Agudelo-Botero

**Affiliations:** 1 Facultad de Estomatología, Universidad Inca Garcilaso de la Vega. Lima, Perú. jorbecerra76@hotmail.com Universidad Inca Garcilaso de la Vega Facultad de Estomatología Universidad Inca Garcilaso de la Vega Lima Peru jorbecerra76@hotmail.com; 2 Instituto Latinoamericano de Altos Estudios en Estomatología (ILAE). Lima, Perú. gfiori@ilaeperu.com Instituto Latinoamericano de Altos Estudios en Estomatología (ILAE) Lima Perú gfiori@ilaeperu.com; 3 Fundación Centro de Investigaciones y Estudios Odontológicos CIEO. Bogotá, Colombia. ortoamariabotero@gmail.com Fundación Centro de Investigaciones y Estudios Odontológicos CIEO Bogotá Colombia ortoamariabotero@gmail.com; 4 Facultad de Estomatología, Universidad Militar Nueva Granada. Bogotá, Colombia Universidad Militar Nueva Granada Facultad de Estomatología Universidad Militar Nueva Granada Bogotá Colombia

**Keywords:** flebolitos, malformaciones vasculares, hemangioma cavernoso, calcificación, phleboliths, vascular malformation, cavernous hemangioman, calcification

## Abstract

Las alteraciones o trastornos vasculares de los vasos sanguíneos o linfáticos presentan características propias, y la identificación de calcificaciones es un parámetro adecuado para realizar un diagnóstico certero. El objetivo del estudio fue describir las características radiográficas de los flebolitos y determinar si representan un desafío para el diagnóstico radiológico. Según la literatura revisada en las bases de datos de Medline (PubMed), SciELO, Google Escolar y algunas revistas especializadas del área, se determinó que los flebolitos tienen características radiográficas muy particulares, de imágenes concéntricas radiotransparentes y radiopacas que semejan a anillos. Sin embargo, no todas las presentaciones son similares, esto hace el diagnóstico engañoso por otras calcificaciones en esta región, como los sialolitos, cuyo aspecto suele ser parecido. A su vez, está la relación con anomalías vasculares y hemangiomas. Finalmente, la evidencia describe características muy propias de esta lesión, pero es necesario individualizar cada caso, por las diferencias que existe de un paciente a otro.

## Abstract (en)

Vascular alterations or disorders of the blood and / or lymphatic vessels have their own characteristics, the identification of calcifications being a correct parameter to be able to make a proper diagnosis. The objective of the study was to describe the radiographic characteristics of the phleboliths, and whether they represent a challenge for diagnosis. according to the literature reviewed in the Medline (PubMed) database, Scielo, Google Scholar and some specialized journals. Phleboliths were found to have very particular radiographic characteristics, with concentric radiolucent and radiopaque images that resemble rings. However, not all the presentations are similar, this makes the diagnosis misleading with other calcifications in this region, such as sialoliths where their appearance is usually similar. Furthermore, there is the relationship with vascular anomalies and hemangiomas. finally, the evidence points to very specific characteristics of this lesion, and it is necessary to individualize each case, due to the differences that exist from one patient to another.

## INTRODUCCIÓN

Los flebolitos son nódulos calcificados idiopáticos poco comunes en la región maxilofacial y se caracterizan por presentar calcificaciones concéntricas radiotransparentes de forma ovalada o circular. Se forman por la acumulación de minerales en un trombo; además, están relacionados con anomalías vasculares (AV), hemangiomas y malformaciones vasculares, o por traumatismos, y se forman por estancamiento vascular ^1^^-^^9^.

Estas AV son alteraciones o trastornos vasculares caracterizados por un desarrollo anormal de los vasos sanguíneos o linfáticos, y la mayoría presentan características propias. La presencia de calcificaciones favorece al camino correcto para el diagnóstico de AV. Se pueden presentar tanto en lactantes como en acianos, pero con frecuencia entre la primera y la tercera década de vida. Aunque no hay predilección por el sexo, se le ha reconocido un alto porcentaje de relación con los músculos masticatorios ^3^^,^^5^^,^^10^^-^^12^.

Para las AV, en 1982, Mulliken y Glowacki plantearon una clasificación biológica basada en su comportamiento, presentación clínica y características patológicas predominantes en el endotelio, y las dividieron en dos grandes grupos: hemangiomas y malformaciones vasculares ^6^^,^^11^^,^^13^^-^^16^.

Los flebolitos relacionados con AV fueron encontrados inicialmente por Constatt, en 1843, en la vena esplénica, y en la región maxilofacial por Kirmission, en 1905. Si bien son muy frecuentes en venas pélvicas, son raras en la región maxilofacial, y en ocasiones se pueden localizar en hemangiomas, pero es patognomónico de las malformaciones vasculares de bajo flujo ^6^.

Los hemangiomas son los tumores más comunes de la infancia y se presentan en el 4 al 10% de los niños, como vasos recién desarrollados de células endoteliales hiperplásicas. Un alto porcentaje aparece en cabeza y cuello, y se diagnostica en el primer año de vida. Se caracterizan por un crecimiento rápido en los primeros meses, seguido por procesos de involución y un desarrollo lento acompañado de más cambios. Un flebolito puede estar asociado a un hemangioma residual de la infancia que podría manifestarse en la adultez ^6^^,^^7^^,^^11^.

Las malformaciones vasculares en la región maxilofacial representan el 40% de todos los casos. La tasa de incidencia es de 1 a 2 casos por cada 10 000 nacidos vivos. Las malformaciones venosas son las más comunes de este grupo y afectan al 1% de la población. Se caracterizan por un desarrollo anormal de las venas con presencia de estancamientos ^15^. Esta condición va a favorecer la formación de flebolitos a nivel vascular, lo que es una característica imagenológica para este tipo de malformación ^4^^,^^6^^,^^7^^,^^17^.

Los exámenes de imágenes cumplen roles importantes para definir el diagnóstico de AV y flebolitos, los datos clínicos del paciente en la historia clínica resultan de mucha ayuda ^3^^,^^10^ y los rayos X de rutina nos dan un diagnóstico inicial. Se presentan como zonas radiopacas, ovaladas concéntricas, cerca de piezas dentales y del ángulo mandibular. La tomografía computarizada (TC) facilita imágenes hipodensas y nítidas de estas calcificaciones, la resonancia magnética (RM) brinda imágenes hiperintensas bien definidas, y el eco Doppler pueden darnos imágenes de pequeños focos hiperecoicos de calcificaciones. Además de evaluar su ubicación, amplitud y límites con estructuras vecinas, todos estos exámenes de imágenes cumplen un rol muy importante para llegar a un consenso en el diagnóstico de estas anomalías y permiten delimitar el camino para su manejo adecuado ^3^^,^^4^^,^^18^^,^^19^. 

Con base en este contexto, el propósito de la investigación se centrará, mediante una revisión narrativa, en describir el diagnóstico de los casos reportados con flebolitos e identificar y conocer sus características imagenológicas en la región maxilofacial con los métodos de imágenes existentes, a fin de que el cirujano dentista tenga la información y el conocimiento básico y especializado de estas calcificaciones para dar un diagnóstico inicial oportuno. 

## MATERIALES Y MÉTODOS

Se realizó una búsqueda bibliográfica en las principales de bases de datos de la literatura científica en ciencias de la salud: Medline a través de PubMed, Google Escolar y SciELO, utilizando las palabras clave “flebolitos”, “malformación vascular”, “hemangioma cavernoso” y “radiografía panorámica". Del mismo modo, se incluyeron artículos en inglés y español publicados hasta el 31de agosto de 2021.

### Etiología, aspectos epidemiológicos y demográficos

Los flebolitos son lesiones poco comunes en la región maxilofacial. Se caracterizan por un alto grado de calcificación y se piensa que se forman por la calcificación de trombos intravenosos ^4^. Su estructura se asemeja a una masa calcificada de forma redondeada, que con frecuencia exhibe una disposición laminar. Su pico alto de crecimiento está dado por la influencia de los fibroblastos y su crecimiento progresa del interior hacia el exterior. Están asociados con lesiones vasculares como los hemangiomas y las malformaciones vasculares ^3^^,^^6^^-^^8^^,^^20^. La disminución del flujo sanguíneo en las AV genera trombos y flebolitos. Su patogenia se transformó a partir de la teoría de Ribbert, quien demostró que los flebolitos aparecen en trombos y se relacionan con las paredes vasculares, donde se mineralizan y desarrollan ^4^^,^^6^.

Su localización o incidencia en cabeza y cuello representa de un 5 a un 20%, con un promedio del 13,5%; en hemangiomas cavernosos, de un 30 a un 50%; y en malformaciones vasculares de tejidos blandos, un 20%. Esta entidad no se asocia con factores hereditarios y lo relacionan más con la falta de apoyo vascular y el estancamiento venoso. Su crecimiento es lento y no presenta sintomatología, se relaciona con los músculos masticatorios y mejilla (27,6%). Aunque son raros en glándulas salivales, se han reportado casos en forma de masas depresibles, edema y, en ocasiones, sintomáticos. Esta calcificación se puede presentar a cualquier edad, de preferencia en la primera y tercera década de vida (55,2%), y no existe predilección por raza o sexo ^3^^,^^4^^,^^6^^,^^7^^,^^15^^,^^21^.

Su incidencia es del 5% al 20% en la región de cabeza y cuello. Su relación con los hemangiomas es del 5% del total de todos los casos de cirugía oral. La región de la mejilla se considera el sitio más común de flebolitos, seguido por las glándulas salivales parótidas, la lengua, los labios y la tiroides. Aunque los flebolitos son raros en glándulas salivales, se han reportado algunos casos. Los flebolitos que se originan en las glándulas salivales pueden causar masas depresibles y edemas que en ocasiones son sintomáticos. La mayoría de estas calcificaciones en la región maxilofacial se encuentran en formación de grupos; por el contrario, los casos raros son solitarios. Una particularidad de la presencia de trombos son los altos niveles de dímero D en sangre, que están presentes en un total del 42% de las malformaciones vasculares. En la actualidad no se han reportado casos de mutación maligna ni de recidivas de flebolitos; sin embargo, es importante hacer seguimiento radiográfico ^4^^,^^6^^,^^9^.

## CARACTERÍSTICAS CLÍNICAS E HISTOLÓGICAS

Los flebolitos son calcificaciones benignas, que comúnmente se presentan como múltiples lesiones cálcicas, rara vez son solitarios y se relacionan con malformaciones vasculares. En la región craneofacial se presentan en un 13,5% y suelen visualizarse en diferentes regiones del paciente y, generalmente, se localizan a través de descubrimientos en exámenes de imágenes de rutina. 

Clínicamente, estas calcificaciones pueden pasar inadvertidas durante mucho tiempo, sin presencia de signos y son evidentes en hallazgos radiográficos. Según la ubicación, se puede presentar en forma de masas blandas o nódulos fluctuantes, de color rojizo o de un color violáceo, algunos de consistencia dura, muestran un desarrollo pausado y limitado. En la mayoría de los casos no se observan síntomas, suelen alterar la imagen facial de los pacientes si la lesión incrementa su volumen ^7^^,^^22^^,^^23^.

Histológicamente, los flebolitos se observan como calcificaciones concéntricas con un patrón laminar con apariencia de cebolla dentro de un vaso, lo que da la apariencia de ojo de buey, y tiene una forma redonda de color marrón rojizo brillante. La lámina externa está formada por tejido conectivo fibroso y de superficie lisa en su interior, que además está compuesta por un tejido blando elástico de color marrón brillante. Este tejido está lleno de glóbulos rojos laminados que forman un trombo organizado y, en el centro de la lesión, hay un cuerpo pequeño parecido a un cálculo incrustado, donde por acciones repetidas va creciendo este tipo de calcificación ^3^^,^^9^^,^^20^^,^^24^^,^^25^.

## DIAGNÓSTICO DIFERENCIAL

La presencia de flebolitos en la región maxilofacial y en tejidos blandos puede ser confusa por la presencia de múltiples calcificaciones, osificaciones y cuerpos extraños que se debe tomar en cuenta en el diagnóstico diferencial. Las calcificaciones son las más frecuentes que se caracterizan por la acumulación de sales de calcio en tejidos y algunas glándulas. Se pueden depositar por mecanismos metastásicos, distróficos o de naturaleza desconocida. Entre las calcificaciones se incluyen entidades como los sialolitos, cálculos calcificados, radiopacos, de forma redondeada y que ocasionan obstrucciones en las glándulas salivales; se caracterizan por el desarrollo de cálculos en la arquitectura ductal glandular o parénquima, se presentan con hinchazón intermitente para estos casos la sialografía sería la herramienta ideal para diagnosticar esta entidad ^3^^,^^5^^,^^16^^,^^20^^,^^24^^,^^26^^-^^28^.

Los nódulos linfáticos calcificados son otra alternativa, y los más afectados son los cervicales y los submandibulares. Se han encontrado también nódulos linfáticos parotídeos debido a procesos inflamatorios crónicos, que es relativamente raro y está asociado principalmente con abscesos necróticos y tuberculosis. La miositis osificante traumática es una entidad de tipo distrófica que puede involucrar a músculos maseteros, pterigoideo medial, esternocleidomastoideo y temporal. Tiene un patrón plumoso a lo largo de las fibras musculares y se dice que es una condición rara causada por traumatismos. También otra patología de confusión es la linfadenopatía, asociada con la tuberculosis, pero que forma un patrón en forma de coliflor en una cadena que la diferencia del resto ^20^^,^^29^.

## ASPECTOS IMAGENOLÓGICOS

Imagenológicamente, los flebolitos se observan dependiendo del tamaño; los más pequeños se pueden apreciar como cuerpos radiopacos de forma redondeada u ovalada, que con frecuencia se localizan a nivel del ángulo mandibular, los de mayor tamaño como cuerpos calcificados de forma redonda u ovoide tienen múltiples laminados que se distribuyen al azar, y de forma circular en el interior con diferenciación de halo radiopaco en el contorno y radiolúcido en el medio, que se asemejan a un ojo de un buey ^5^^,^^24^. En ocasiones, tiene un núcleo radiopaco o radiolúcido, y la repetición de estas calcificaciones tiene una apariencia de anillos, su presentación es múltiple; sin embargo, se han reportado casos únicos. Se presenta con mayor frecuencia en las mejillas, seguido de la parótida y otros tejidos bucales ^3^^,^^5^^,^^16^^,^^24^^,^^25^^,^^28^^,^^30^.

La tomografía computarizada (TC), por su sensibilidad, es otra herramienta clave para el diagnóstico de tejidos duros y calcificaciones, como es el caso del flebolito y demás afecciones. En esta se observa como múltiples imágenes hiperdensas circulares u ovalados con diámetros diferentes distribuidas en grupos al azar; en ocasiones pueden ser únicos ^6^^,^^7^^,^^28^.

La resonancia magnética (RM), más que para la detección de flebolitos, es una herramienta para la detección de tejidos blandos teniendo en cuenta zonas superficiales y profundas ^28^, y en este caso en especial tumores y malformaciones vasculares que están relacionadas con los flebolitos. Su principal característica es la presencia de cavitaciones y vacíos de forma redondeada u ovalada, zonas hiperintensas sin vacíos de flujos que confirman la característica de los flebolitos. Finalmente, podemos mencionar que además de los exámenes auxiliares por imágenes una biopsia seguida de un estudio microscópico ayudaría al diagnóstico final ^28^^,^^31^.

**Figura 1:**
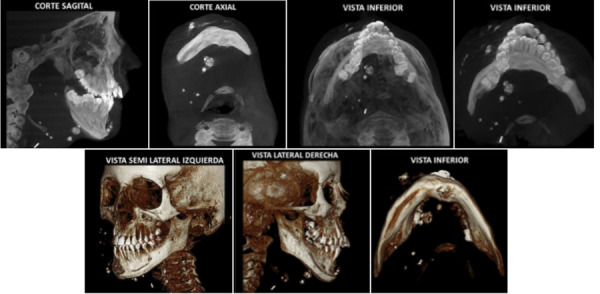
Múltiples calcificaciones en región maxilofacial, flebolitos. Cortesía del Dr. Hugo Aguayo del Centro de Diagnóstico por Imágenes (CDI), Lima, Perú.

Como datos históricos adicionales se puede mencionar que los métodos diagnósticos han evolucionado con el tiempo, y muestra de ello se observa que los exámenes de imágenes y equipos de diagnóstico han superado estándares antes pensados, lo que brinda tecnología avanzada por el bien de la salud. Al ser así, la literatura reporta múltiples casos en los últimos años, que resaltan estudios de imágenes más digitalizados y tecnificados que en años anteriores al milenio (Figura 1, Tabla 1).

**Tabla 1:** Descripción de flebolitos reportados en la literatura científica en este nuevo milenio

	Año	Edad	Sexo	Localización	Tamaño (mm)	Dolor	Edema	Imagen	Tratamiento
Unal ^32^	2003	18	M	Sublingual	25x15	-	+	TC RM	Cirugía
Scolozzi ^16^	2003	92	F	T. blando	1-6	+	-	2D	Ninguno
Chuang ^33^	2005	65	M	Submandibular		+	+	TC	Enucleación
Altug ^34^	2007	21	M	Masetero	4X5	+	+	TC	Ninguno
		22	M			-	+	TC 2D	Ninguno
		21	M	SCM		-	+	TC	Ninguno
Su ^35^	2009	23	F	Submandibular		+	+	2D R	Escleroterapia
Mandel ^8^	2010	56	F	Bucal		-	+	2D TC	Ninguno
		64	F	Lengua		-	+	2D	Embolización
Mohan ^36^	2011	45	F	Mucosa	2x2	-	+	TC R	Ninguno
Kato ^20^	2012	17	F	Masetero	17x14	-	+	TC	Cirugía
Orhan ^37^	2012	20	M	Múltiple			+	TC	Embolización
Zengin ^28^	2013	21	F	Masetero	2-10	-	+	RM	Ninguno
Choi ^38^	2013	44	M	Parótida	60x40	-	+	TC	Cirugía
Chava ^39^	2013	28	M	Múltiples	30x20	-	+	RM	Ninguno
Gooi ^1^	2014	14	F	Submandibular	8x7	-	-	TC RM	Ninguno
		7	F	Submandibular	33x42	-	+	TC RM	Escleroterapia
Aynali ^40^	2014	7	M	Submandibular	4-8	-	+	TC	Enucleación
Kamatani ^9^	2015	51	M	Lengua			+	TC RM	Cirugía
Lima ^3^	2015	56	F	Labio	3	-	+		
Ghosh ^23^	2015	37	M	Múltiple		-	+	RM AN	Ninguno
Nagaraja ^41^	2016	49	M	Bucal	25x15	-	-	2D R	Cirugía
Fernández ^6^	2016	9	M	Parótida		-	+	TC 2D	Escleroterapia
Chen ^42^	2017	43	F	Parótida	50	-	+	TC	Cirugía
Prakash ^43^	2017	30	M	Múltiple	30x40	+	+	2D CD	Ninguno
Arroyo ^7^	2018	18	M	R mentoniana	40x50	-	+	TC	Cirugía
		17	F	Masetero	35x40	-	+	TC	Cirugía
		17	F	R mentoniana	3x4	-	+	TC	Cirugía
Sivrikaya ^2^	2019	26	M	R bucal	32	-	+	2D TC	Ninguno
Shinichi ^4^	2020	66	F	A mandibular			+	2D RM	Ninguno

Fuente: Literatura universal

## CONCLUSIONES

A pesar del índice bajo de calcificaciones en la región maxilofacial, los cirujanos dentistas deben conocer la existencia de estas entidades, y familiarizarse con las características radiográficas que presentan; del mismo modo, el diagnóstico diferencial, que causa controversias en su interpretación y diagnóstico. La búsqueda de información da a conocer que los flebolitos en la región maxilofacial son poco comunes, y se relacionan con hemangiomas y malformaciones vasculares, cabe destacar que estas entidades son identificadas como hallazgos radiográficos de rutina y que en diferentes circunstancias pasan por alto. Por lo tanto, es competencia del radiólogo maxilofacial reportar en sus informes estas calcificaciones, para que el clínico este familiarizado, y así informar a sus pacientes de la existencia de estas entidades y orientar como se debe actuar o remitir a la instancia correspondiente para su atención oportuna.

## Fuente de financiamiento:

Autofinanciado
